# Utilization of *Allium* peels to improve soil water holding capacity and rice growth under water stress conditions

**DOI:** 10.1038/s41598-025-27336-8

**Published:** 2025-11-25

**Authors:** Elham R. S. Soliman, Basant M. Ahmed, Jalla H. Mohamed, Reda E. Abdelhameed

**Affiliations:** 1 Botany and Microbiology Department, Faculty of Science, Helwan University, Cairo, 11795 Egypt; 2 Molecular Biotechnology program Chemistry Department, Faculty of Science, Helwan University, Cairo, 11795 Egypt; 3 Botany and Microbiology Department, Faculty of Science, Zagazig University, Zagazig, 44519 Egypt

**Keywords:** Agricultural wastes, Garlic peels, Genome integrity, Non-protein thiols, Water holding capacity, Water stress, Biotechnology, Physiology, Plant sciences

## Abstract

Water scarcity hampered rice production and jeopardized worldwide food security. Under conditions of water scarcity, this research investigates the effect of four different *Allium* peels on soil water holding capacity (WHC) and rice seedlings development. Physicochemical analysis of red, yellow onions, and garlic peels was performed, and their application for growing rice in soil irrigated with 50% field capacity compared to the well-irrigated control group was achieved. Garlic peels contained the least sulphur (0.007 g/g), while red onion peels contained the most (0.147 g/g). The contents of crude fiber, ash, carbohydrates, proteins, phenolic, and flavonoids varied between species. The application of garlic peel to soil resulted in a 60% increase in the soil WHC, which was directly proportional to the fiber content of each peel. Rice seedlings cultivated in pots supplemented with different peels, especially the garlic peel, exhibited substantial growth when subjected to restricted water regimes. Rice plant pigment fractions and hydration status were drastically reduced due to water constraints; however, the addition of garlic and onion peels supplements mitigated this effect. Most especially, supplementing soil with garlic peels caused an enhancement of 66.7% and 80.22% in length and fresh weight of shoot, 59.9% and 87.3% in chlorophyll a and b. The carotenoids, water content, proline, protein, enzymatic antioxidants, and non-protein thiols were all increased by these peels, while rice leaf oxidative impairment (lipooxygenase, lipid peroxidation, and H_2_O_2_) was decreased. ISSR analysis detects a positive correlation between genomic profiles of plants cultivated in different peel-amended soils. Agro-waste may support soil management, promote rice growth in conditions of limited irrigation water, and provide solutions for water scarcity and waste management. Water conservation and sustainable agriculture are made possible by this novel method.

**Supplementary Information:**

The online version contains supplementary material available at 10.1038/s41598-025-27336-8.

## Introduction

Concurrent with the incremental increase in population is the rising demand for agricultural products to sustain the expanded populace. This resulted in an upsurge in the production of agro-waste, which has strained or even disrupted numerous environmental system components. These agro-wastes derived from fruits and vegetables are an excellent source of interesting compounds based on their composition. They can be applied in various technological contexts, adding value to the industrial, agricultural, and medical fields^1,2^. Consequently, there is a global recognition of the critical nature of establishing a suitable and ecologically sound management approach for agricultural byproducts. Reuse and recycling of these waste materials are considered the most favorable methods in terms of ensuring long-term economic sustainability^3^.

Following tomatoes, onion species are the second most significant horticultural vegetables that account for about 13% of world vegetable production increase during 2000 and 2019^4^. Recent increases in processed onion demand have resulted in a corresponding rise in the amount of onion waste generated. Onion skins, two outer fleshy scales, roots produced during industrial peeling, and bulbs that are under-sized, malformed, diseased, or damaged, constitute the majority of onion waste. The environmental impact of these waste materials is concerning. Onion waste has the potential to be valorized as a supplier of useful components due to its complex and intriguing chemical composition and well-established health-promoting qualities^1,5,6^. Contrary, onion wastes, at high concentrations, are unsuitable for fodder due to the pungent aroma they emit. Additionally, due to the fast development of phytopathogenic agents, they cannot be used as an organic fertilizer^5,7^. The composition of onions exhibits variability based on factors such as cultivar, maturation stage, storage duration, bulb section, and environmental and agrotechnical conditions^7^.

Garlic and onions possess an exceptional abundance of organosulfur compounds, which contribute in part to its advantageous impact on health and provide the distinctive aroma and flavor of onions and garlic^8^. Carbohydrates, organosulfur compounds, protein, free amino acids, vitamins, trace elements, and total fibers are among the numerous nutrient components found in garlic and onions^7,9,10^. Antioxidant, anticancer, cholesterol-lowering, and immune-enhancing, antimicrobial, and anti-inflammatory properties have been extensively documented in human and animal studies of garlic and onions^8,10–12^. A chromatographic evaluation of the proteins isolated from the outer and inner skins of garlic identified 67 proteins in total^13^. The biochemical and physiological characteristics of these compounds provide opportunities for their continued utilization as functional food constituents, which can improve the antioxidant and prebiotic properties of innovative products^7,9^.

The primary limitation that continues to impede sustainable agricultural production is the dearth of water^14,15^. A shortage of water restricts the availability of nutrients and disrupts physiological processes, which lowers agricultural productivity^16^. By 2050, global crop productivity is expected to drop by 9% due to climate change, as increased global warming is expected to increase the frequency of drought occurrence^16^. This phenomenon (water shortage; WS) leads to an increased reliance on substitute water sources, such as treated municipal water and agricultural drainage water, for irrigation purposes^17,18^.

Soil water holding capacity (WHC), which is defined as the overall water retention capacity of a soil after drainage of excess water, is critical for crop production and mitigates the unfavorable consequences of climate change by providing a buffer for yield fluctuations caused by weather variations^16,19^. Agro-ecosystem adaptation to the advent of catastrophic occurrences like drought, is facilitated by an increase in soil WHC, which strengthens the soil’s resistance to the fluctuating climate. Consequently, increasing soil WHC through environmentally friendly and sustainable methods is an imperative necessity^20^. Furthermore, it has been documented that the incorporation of organic matter into soil to sequester carbon can contribute to reducing climate change^20,21^. In this respect, the augmentation of soil organic carbon (OC) has the potential to enhance soil quality through various mechanisms. These include but are not limited to promoting aggregation and structural enhancement, bolstering biological activity of soil, and optimizing soil moisture and temperature^22^. Additionally, high soil OC is recommended for drought management^21^. Thus, the use of organic amendments serves as a source of carbon and other nutrients that support WHC and improve soil structure, fostering plant growth while reducing the need for chemical products for crop fertilization and the ongoing degradation of land^23^.

Rice ranks among the world’s most important grain crops, cultivated widely across diverse regions and climatic conditions throughout the year. While the grains serve primarily as a staple food for human consumption, other plant parts are used as animal feed and for various domestic purposes by farmers. It is the primary food source for nearly half of the planet’s population^24,25^, and no other major crop supplies as many people with such a significant share of their daily caloric intake. Beyond calories, rice provides essential nutrients, including magnesium, phosphorus, manganese, selenium, iron, folic acid, thiamin, and niacin, although it is naturally low in fiber and fat. Also, it contains phenolic compounds such as phytic acid and phenols, along with sterols, flavonoids, terpenoids, anthocyanins, tocopherols, tocotrienols, and oryzanol. These bioactive components are associated with strong antioxidant activity and have been linked to reducing the risk of cardiovascular disease and diabetes. Incorporating rice into a balanced diet can help sustain energy, promote healthy digestion, and support brain function, making it a beneficial component of everyday nutrition^26,27^. Consequently, the current study’s goal is to use *Allium* peels as an alternative to enhance soil WHC and improve rice growth under a restricted water regime. Therefore, an *in vivo* experiment to grow rice seedlings in soil amended with different *Allium* peels was performed, and the vegetative growth parameters, physiological, biochemical, and genetic attributes of these plants were evaluated.

## Materials and Methods

### Biowaste samples’ preparation

Red (Beheri cultivar) and yellow (Giza 20 cultivar) onions (*Allium cepa* L.)^28^ and garlic (*Allium sativum* L. Balady cultivar)^29^ were purchased at the ripe stage from a local market in Egypt (August 2023). The outer dry protective layers of the onion and garlic were separated, thoroughly cleaned with tap water to get rid of any dust and other contaminants, opened in a permeable tray to drain extra water, and then air-dried. Then grounded individually or in a 1:1:1 ratio (mix) in an electric grinder to a powder and stored in clean, dry glass jars at room temperature till use.

### The water extract’s preparation

Water extract of the peels (red onion, yellow onion, garlic, and mix) was prepared according to Chia et al.^30^. To sum up, 5 g of every peel powder was mixed with 100 mL of distilled water, then incubated at 70 °C for 3 h. Then the mix was filtered using filter paper (Whatman #2), after that, the filtrate was kept at −20 °C until needed.

### Physicochemical analysis of the peels

The ash content was determined in accordance with Park et al.^31^, by placing 1 g of each peel sample in a muffle furnace (STUART SCIENTIFIC, United Kingdom) at 350 °C for 3 h for ashing. After cooling down, the ash content was weighed using a digital balance. The total fiber content was measured according to AOAC International^32^. One gram of each sample was digested by boiling for 15 min in 0.128 M sulfuric acid, followed by another 15 min of boiling in 0.313 M sodium hydroxide solution. The collected fibers were dried in an oven at 230 °C for 2 h, then weighed. Carbohydrate content was calculated according to Loewus^33^ while the sulphur content was evaluated using an adjusted methodology of Massoumi and Cornfield^34^ and its concentration was quantified as mg of sulphur equivalent per gram of dry weight (DW) using a standard curve.

Protein content of the peels was quantified according to Lowry et al.^35^, by mixing 1 mL of peel’s water extract with 5 mL of sol C (50 mL sol A {2% Na_2_CO_3_ in 0.4% NaOH} +1mL sol B {0.5% CuSO_4_ in 1% sodium tartrate}), then shake well, incubate it for 10 min at room temperature. After that, 0.5 mL of Folin reagent was added, the mixture was thoroughly shaken, and it was allowed to sit at room temperature for 30 min before the absorbance at 750 nm was measured. The protein content was quantified as mg of Bovine Serum Albumin (BSA) equivalent per gram of DW. Total phenol was measured following the method of the Folin-Ciocalteau assay^36^, 1 mL of each water extract was combined with 2.5 mL of 1/10 dilution of Folin Ciocalteaus reagent and 2 mL of Na_2_CO_3_ (7.5% w/v), incubated at 45 °C for 25 min then measured at 765 nm with a spectrophotometer (CECIL; CE1010, England). The concentration was expressed as mg of gallic acid equivalent (GAE) per gram of DW.

Total flavonoid contents of the peels were determined by the aluminum chloride colorimetric method^37^. 200 µL of the water extract was mixed with 300 µL of 5% NaNO_2_ and allowed to stand for 5 min at room temperature. Then 600 µL of 10% ACl_3_ was added, after 6 min, 2 mL of 1 M NaOH, and 2 mL of dist. water was added. The solution was incubated at 25 °C for 15 min then the absorbance of the samples was measured at 510 nm. The concentration was expressed as mg of quercetin equivalent (QE) per gram of DW. The total antioxidant capacity (TAC) was measured using the method of Prieto et al.^38^, 0.3 mL of peel extract was combined with 3 mL of the following reagent (0.6 M sulphuric acid, 28 mM sodium phosphate, 4 mM ammonium molybdate). The samples were incubated at 95 °C for 90 min, then allowed to cool. The absorbance of the samples was measured at 695 nm, and the TAC was expressed as µg ascorbic acid equivalents/g DW.

### Soil water properties with the peels

#### Moisture content

Moisture content was measured according to the gravimetric method^39^. Five grams of coarse sandy soil was weighed, then oven dried at 105 °C for 24 h (till a constant weight is obtained). The moisture content was calculated by measuring the difference between the fresh weight and the oven-dry weight. Each type of peels was mixed with the soil in a ratio of 1:10 (w/w), respectively, and the aforementioned steps were applied to calculate the effect of mixing the peels with the soil on its moisture content.

#### Determination of water holding capacity (WHC)

The WHC was calculated using the gravimetric method according to Nigam et al.^40^. Five grams of coarse sandy soil were placed in a separate funnel, lined with filter paper. The funnel was positioned over a measuring cylinder to collect excess water. 50 mL of water was slowly poured over the soil. The saturated soil was weighed to calculate how much water the soil absorbed. The sandy soil was used as it is well known that it has very low WHC. To confirm if mixing the soil with the *Allium* peels would increase the WHC of the soil. Each type of peel was mixed with the soil in a ratio of 1:10 (w/w), respectively, and the aforementioned steps were applied. The percent of WHC was computed using the following formula:

1 $$\:\%\:of\:WHC=\frac{(\text{W}\text{t}\:\text{o}\text{f}\:\text{s}\text{a}\text{t}\text{u}\text{r}\text{a}\text{t}\text{e}\text{d}\:\text{s}\text{o}\text{i}\text{l}-\text{W}\text{t}\:\text{o}\text{f}\:\text{d}\text{r}\text{y}\:\text{s}\text{o}\text{i}\text{l})}{\text{W}\text{t}\:\text{o}\text{f}\:\text{d}\text{r}\text{y}\:\text{s}\text{o}\text{i}\text{l}}\:X\:100.$$

### Experimental design, *in vivo* plant growth condition and treatments

Giza 179 rice seed cultivars^41^ were generously donated by the Agriculture Research Centre in Giza, Egypt. Following a dust-free rinse with running tap water, the seeds were soaked in fully saturated paper tissue for four days, till the radicles emerged. For the control group, 15 seeds were sown in triplicate in sterilized plastic pots with a diameter of 10 cm. These pots were filled with 0.5 kg of sterile soil, which was a 2:1 mixture of sand and peat moss (v/v). The analysis of the soil using sieve methods revealed particle size distribution as follows: 5% > mm, 11.8% ˂2 mm, 19.15% ˂0.2 mm, 52.8% ˂0.02 mm, 7% ˂0.002 mm, and 3.7% ˂0.0002 mm with a pH: 8.40 and electrical conductivity (EC: 0.58 dS/m). The soil contained 24.6 ppm (P), 0.72 ppm (Fe), 0.18 ppm (Cu), 0.02 ppm (Mn), 21.84 ppm (Mg), and 203.8 ppm (K) as detected using a Microwave Plasma Atomic Emission Spectrometer, Agilent Inc. The remaining groups are cultivated in soil amended with peels, wherein a 3:1 (soil mix: each peel type (v/v)); (red onion, yellow onion, garlic, or a combination thereof). Up until they were ten days old, the plants were consistently irrigated with an equivalent quantity of water every three days until the soil was completely saturated. Then the control group was divided into two subgroups named: control (continuously watered with water amount to ensure full saturation, 100%).The second subgroup, referred to as water-stressed (water shortage, WS), was irrigated with 50% field capacity. All of the seedlings sown in soil amended with peels exhibited water stress, as the plants were irrigated with only 50% field capacity. A 50% field capacity was maintained by irrigating each pot to 50% of its field capacity, calculated based on the difference between pot weight at full saturation and oven-dry weight, and replenished as needed throughout the experiment. Collectively, there are 6 treatments (Table 1) with 3 replicas for each treatment, giving 18 pots used for planting in a randomized complete block design. The plants continue to be hydrated as described earlier until they reach 30 days of age. At this age, samples were taken for measuring vegetative growth and other analyses. In order to do additional physiological and biochemical analysis as well as genetic profiling, the sampled leaves were washed using water, immediately put in liquid nitrogen, and kept in a freezer at a low temperature (−20 °C) till use.

**Table 1:** Treatments used in the experimental design of the *in vivo* assay.

Treatments	Explanation
**Control**	Continuously watered with water to ensure full saturation, 100% field capacity
**WS**	Water-stressed (water shortage): irrigated with 50% field capacity
**Red**	Red onion peels amended the soil and exhibited water stress, as the plants were irrigated with only 50% field capacity
**Yellow**	Yellow onion peels amended the soil and exhibited water stress, as the plants were irrigated with only 50% field capacity
**Garlic**	Garlic peels amended the soil and exhibited water stress, as the plants were irrigated with only 50% field capacity
**Mix**	Mix (1:1:1) of red, yellow, and garlic peels amended soil exhibited water stress, as the plants were irrigated with only 50% field capacity

#### Vegetative growth attributes

Randomly harvested plants from all treatments were separated into roots and shoots. Shoot and root length (cm) was measured straightaway after the harvesting using a graduated ruler, and the fresh weight (FW; g) of the shoots and roots was measured using a digital balance. Data for shoot and root dry weights (DW; g) were estimated following oven-drying at 65 °C for 48 h.

#### Measurement of physiological traits of rice seedlings

##### Chlorophyll and carotenoids concentrations

According to Metzner et al.^42^, measurements of carotenoids, chlorophyll a, and b were made in rice leaf tissue samples in acetone (85%). The plant material that had been homogenized was then centrifuged at 6000 g. The absorbance at 663, 644, and 452 nm was measured using a spectrophotometer then were stated as mg/g FW using the equations below:

2 $${\text{Chl a }}={\text{ }}\left[ {12.7\left( {{\text{A}}663} \right){\text{ }}-{\text{ }}2.69{\text{ }}\left( {{\text{A}}644} \right)} \right]{\text{ }} \times {\text{ V}}/1000{\text{ }} \times {\text{ FW}}$$

3 $${\text{Chl b }}={\text{ }}\left[ {22.9\left( {{\text{A}}644} \right){\text{ }}-{\text{ }}4.68{\text{ }}\left( {{\text{A}}663} \right)} \right]{\text{ }} \times {\text{ V}}/1000{\text{ }} \times {\text{ FW}}$$

4 $${\text{Carotenoids }}={\text{ }}\left( {4.2{\text{ }}A452.5} \right){\text{ }}-{\text{ }}\left( {0.0264{\text{ Chl}}{\text{. a}}\,+\,0.426{\text{ Chl}}{\text{. b}}} \right){\text{ }} \times {\text{ V}}/{\text{ }}\left( {1000{\text{ }} \times {\text{FW}}} \right)$$

*A = absorbance, V = volume of sample, FW = Fresh weight of sample.

##### Leaf water content

According to the protocol outlined by Barrs and Weatherley^43^ measurements of water content (WC), relative water content (RWC), and water saturation deficit (WSD) were taken. Following harvest, the fresh weight (FW) of the rice leaves was established, and ten leaf discs (1–2 cm diameter) were subsequently allowed to float on distilled water, fully submerged in the dark until fully rehydrated. Then, samples were removed after 4 h, surface water was eliminated, and samples were once more weighed to achieve the fully turgid weight (TW). In order to achieve a consistent weight, the turgid leaves were dried in a hot air oven at 60 °C, and the dry weight (DW) was noted. WC, RWC, and WSD were recorded utilizing the following equations:

5 $$\:WC=\frac{FW-DW}{FW}X100$$

6 $$\:RWC=\frac{FW-DW}{TW-DW}X100$$

7 $$\:WSD=100-RWC$$

#### Measurement of biochemical parameters

##### Soluble protein and free proline contents

The total soluble protein of fresh rice leaves under different treatments (0.25 g) was homogenized with 5 mL of potassium phosphate buffer pH 7.0. The homogenate was then centrifuged (6000 g) for 30 min at 4 °C. The protein concentration was assayed and calculated according to the Lowry protocol ^35^ as previously mentioned above, then expressed as mg/g FW.

The method described by Bates et al.^44^, was used to estimate free proline. Ten mL of 3% aqueous sulfosalicylic acid was used to grind up around 0.5 g of fresh leaf. For an hour, two mL of the extract and two mL of the acid ninhydrin reagent were combined and stored at 100 °C. Toluene (4 mL) was used to extract the proline, then toluene was cleared from the aqueous phase at room temperature. On a spectrophotometer set at 520 nm, the absorbance was evaluated, and proline content was assayed using a standard curve for L-proline. As µmol g^− 1^ FW, the proline is expressed.

##### Lipid peroxidation (expressed as malondialdehyde content, MDA)

Using Dhindsa et al.^45^, the generation of MDA was used to determine lipid peroxidation. 250 mg of fresh leaves were homogenized in 5 mL of 0.1% (w/v) trichloroacetic acid, and they were centrifuged for 10 min at 8000 rpm. Next, 2 mL of 20% tichloroacetic acid containing 0.5% (/v) thiobarbituric acid was combined with 2 mL of the supernatant. After 30 min of incubation in a bath of boiling water, the solution was chilled on ice. After centrifuging the extract once again for five minutes at 8000 rpm, the mixture’s absorbance was measured at 532 and 600 nm using a UV/VIS spectrophotometer, and the MDA content was identified as µmol g^− 1^ FW.

##### H_2_O_2_ content

Using a spectrophotometer, the H_2_O_2_ content was measured following Velikova et al. ^46^ methodology. 250 mg of rice leaf samples were homogenized in trichloroacetic acid under various treatment conditions. For fifteen minutes, the homogenate was centrifuged at 8000 g. Then, 0.5 mL of the supernatant was combined with 2 mL of potassium iodide (1 M) and 0.5 mL of 10 mM potassium phosphate buffer (pH 7.0). Each sample’s H_2_O_2_ content was calculated as mg g^− 1^ FW by comparing its absorbance at 390 nm to a standard calibration curve.

##### Total thiol and non-protein thiol

In conformity withSedlak and Lindsay ^47^ procedure, the levels of total thiol and non-protein thiol in fresh rice leaves were determined using Ellman’s reagent. Total sulfhydryl groups were calculated and given as mg/g FW using an extinction coefficient of 13,600. Non-protein thiol concentration was evaluated in 2 mL of deproteinized supernatant, similarly to the total thiol concentration.

##### Enzymatic activity assays

Enzymes were extracted according to Qiu et al.^48^. One gram of fresh rice leaves was homogenized in 10 mL of ice-cold extraction solution containing 50 mM potassium phosphate buffer (pH 7), 0.1 mM ethylenediaminetetraacetic acid (EDTA), and 1% (w/v) polyvinylpyrrolidone. The homogenate was centrifuged at 8000 g for 20 min at 4 °C. The supernatant was collected for enzymatic tests.

- **Activity of catalase (CAT)**: CAT (EC 1.11.1.6) activity was determined by the consumption of H_2_O_2_^49^ where a 3 mL reaction mixture containing 2.5 mL of 0.1 M phosphate buffer (pH 7.0), 0.2 mL supernatant, and 0.3 mL of 100 mM H_2_O_2_ was added sequentially, and the absorbance was measured at 240 nm.
- **Activity of peroxidase (POX)**: With slight modifications, the activity of POX (EC 1.11.1.7) was assessed using the methodology of **Chance and Maehly**^50^. The reaction mixture consisted of 0.33 mM pyrogallol, 10 mM potassium phosphate buffer (pH 7.0), and 0.5 mL of enzyme extract. The reaction was initiated by adding 40 mM H_2_O_2_ and the absorbance was measured at 470 nm.
- **Activity of polyphenol oxidase (PPO) and ascorbate peroxidase (APX)**: The activities of PPO (1.14.18.1)^51^ and APX (EC 1.1.11.1)^52^ were measured in the supernatant and expressed as U g^− 1^ FW. An assay of PPO was performed, wherein 5 mL of assay mixture comprising 125 µM of phosphate buffer (pH 6.8), 100 µM of pyrogallol, and 1 mL of enzyme extract were prepared. The optical density of the produced color was measured at 430 nm. APX was measured in a reaction mixture containing 2.5 mL of 0.1 M phosphate buffer (pH 7.0), 0.1 mL L-ascorbate, and 0.15 mL H_2_O_2_. The reaction was started by adding 250 µL of enzyme extract. A decrease in absorbance at 290 nm was measured spectrophotometrically compared to a blank.
- **Activity of lipooxygenase (LOX)**: By measuring the absorbance at 234 nm, LOX (EC 1.13.11.12) activity in rice extract was determined^53^. The reaction was initiated by adding 0.25 mL of crude extract to 2 mL of a 50 mM sodium phosphate buffer (pH 7.5) containing 1 mM of linoleate.

#### Effect on the genetic profiling of rice seedlings

Genomic DNA was extracted from rice seedlings using CTAB DNA extraction protocol^54^. The isolated DNA was resolved on a 1.2% aarose gel and electrophoresed in 1× TAE buffer containing 0.5 µg/mL ethidium bromide in order to confirm the integrity of the DNA. Using the primers provided in Table 2in a 25 µL reaction volume, the polymerase chain reaction (PCR) was carried out in a Biometra thermal cycler. As instructed by Soliman et al.^55^, the PCR and cycling were done. The PCR reaction was carried out using *Cosmo-Taq* DNA polymerase (Willowfort: WF-1020201), following the manufacturer’s instructions. The amplified PCR products were resolved in a 1.2% agaroe gel containing 0.5 µg/mL ethidium bromide in 1× Tris-acetate-ethylenediaminetetraacetic acid (TAE) buffer. The resolved bands were visualized using a UV-transilluminator (made by Vilber Lourmat in Germany). The Inter-Simple Sequence Repeats (ISSR) markers were analyzed using Quantity One Software 4.6.2.70 for the generation of binary data to score the band’s existence, with 1 and 0 denoting absence. For each primer and treatment, the total number of bands as well as the percentage of polymorphism were calculated. Thermo Scientific #SM0241’s GeneRuler 100 bp DNA ladder was used to measure the size of the ISSR fragments.

**Table 2:** List of selected ISSR primers, including their codes, sequences, annealing temperature, number of amplified markers, and percentage of polymorphism (changes in genetic content) for each primer in each treatment.

No.	Primers codes	Sequences (5′−3′)	Annealing temp. (°c)	No. of Polymorphic markers	Total no. of markers	% of polymorphism
1.	HB14	(GT)_6_CC	50	2	4	50
2.	I-885	CGTACTCGT(GA)_5_		2	3	66.7
3.	I-889	AGTCGAGT(AC)_5_		1	3	33.33
4.	I-891	ACTACGACT(TG)_5_T		7	9	77.8
5.	ISSR-5	(ACG)_4_GAC		2	3	66.7
Total				14	22	63.6
Control				19	-	86.4
Water stressed (WS)				14	-	63.6
Garlic peels				19	-	86.4
Red onion peels				12	-	54.54
Yellow onion peels				14	-	63.6
Mix peels				16	-	72.72

### Statistical data analysis and drawing figures

For every treatment, the information is displayed as means ± standard error (SE) of six replicates. Duncan’s post hoc test is conducted after one-way analysis of variance (one-way ANOVA) for the data using the SPSS program. Lowercase letters (a, b, c, and d; *p* ≤ 0.05) were also used to indicate the statistical significance of the mean value comparison. Principal Component Analysis (PCA; biplot), Pearson correlation, and Hierarchical Clustering Analysis (HCA) between different treatments were performed using the PAST-pc.

## Results

### *Allium* peels: a physicochemical examination

**Fig. 1:**
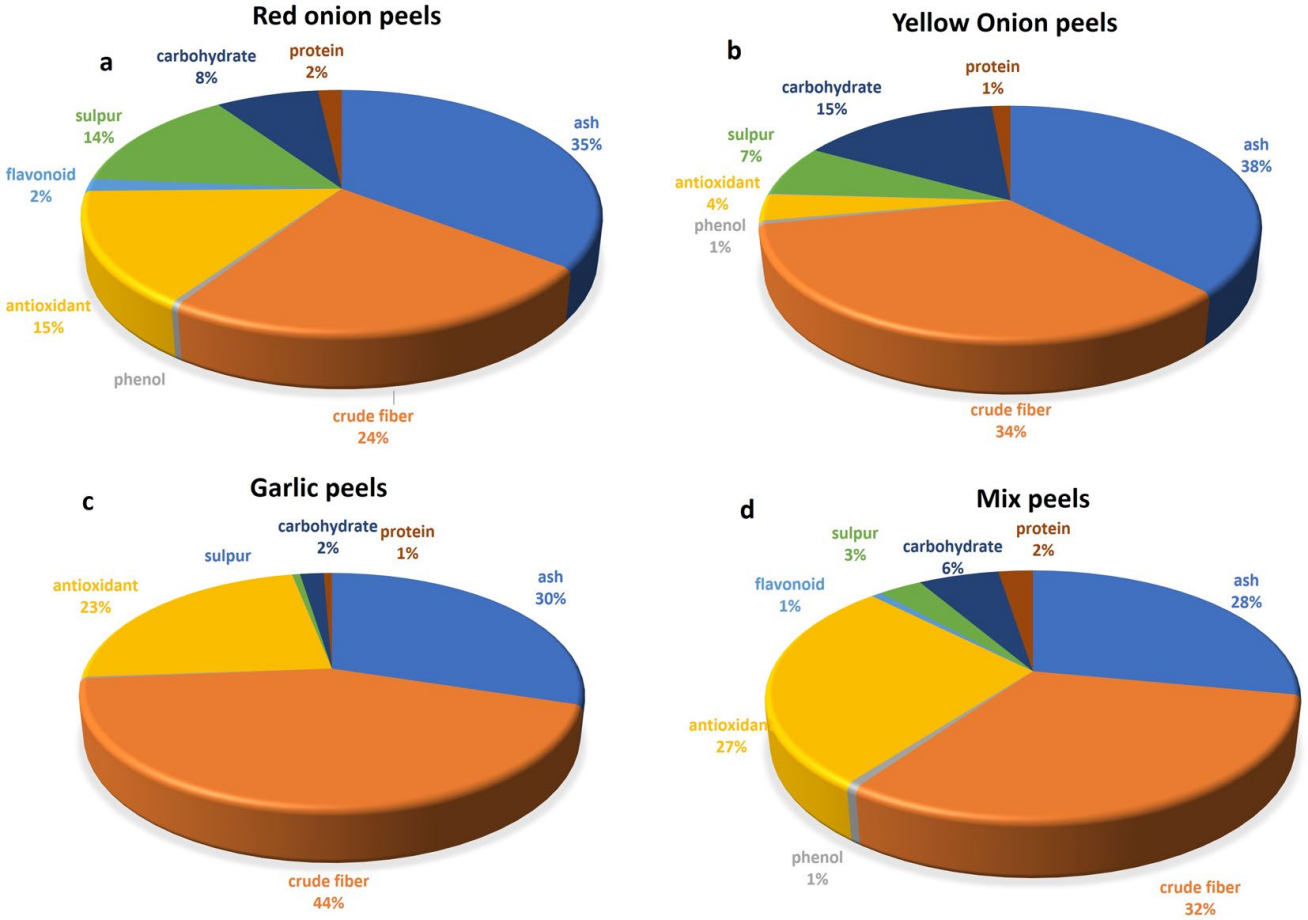
The physicochemical properties of the biowaste materials: **a**: red onion peels, **b**: yellow onion peels, **c**: garlic peels, and **d**: mixed peels.

The bulk components of the *Allium* peels utilized in the present investigation, as determined by physiochemical analysis **(**Fig. 1**)**, consist of fibers and ashes, which account for 28 to 38% of the peels’ total composition. The highest concentration of antioxidants was 0.3 mg/g DW in the garlic peel, representing 23% of the total contents, followed by mixed peels and red onion peels (0.18 and 0.16 mg/g DW, respectively). Onion and garlic scale leaves contain a considerable amount of phenolics, the least in garlic (2.87 ± 0.031 mg GAE/g DW) and the highest (4.51 ± 0.08 mg GAE/g DW) in red onion peels were detected. While flavonoid concentration was varied, however in overall it represents ≤ 2% of the total composition in all *Allium* peels of a maximum of (17.48 mg QE/g DW) in red onion peel powder. The higher amount of sulphur was detected in red onion peels, representing 14% while only 0.56% was detected in garlic peels.

## Effect of *Allium* peels on the water holding capacity of the soil

**Fig. 2:**
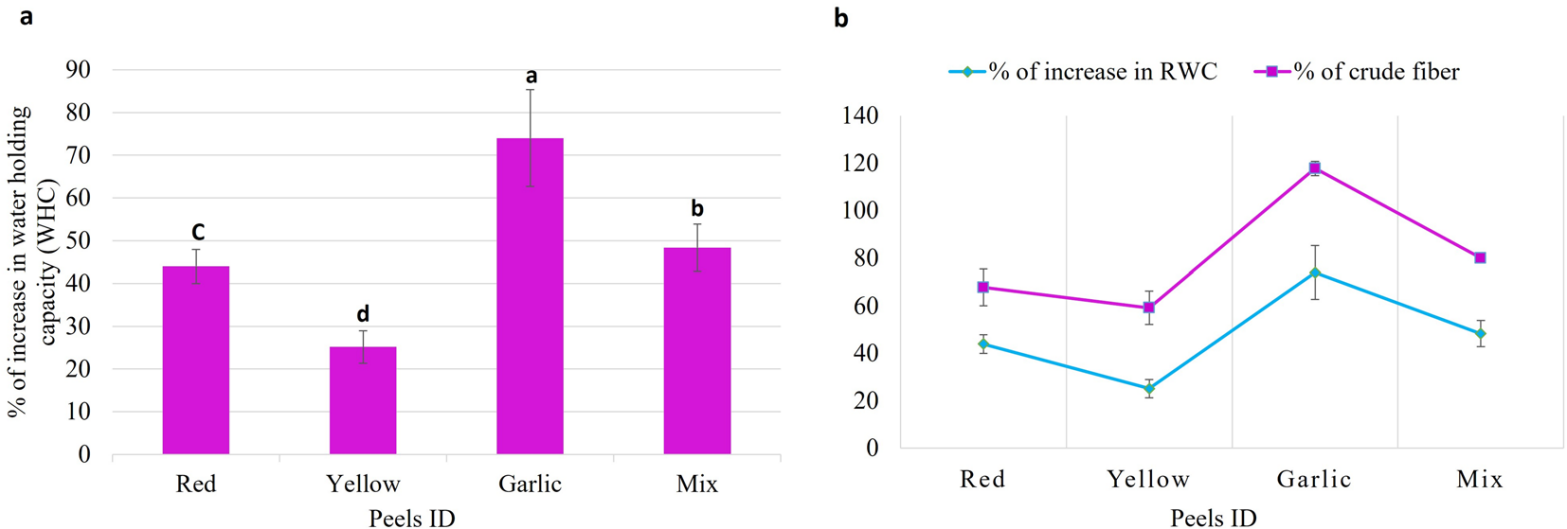
Effect of *Allium* peels on water holding capacity (WHC) of the soil **a**: Percent of increase in the water holding capacity (WHC) of the soil upon mixing with different peels. The data represent the average of three independent replicas with *p* value = 0.000127. **b**: A stacked line chart shows how the percentage of increase in the RWC of the soil is proportional to the amount of fibers in different *Allium* peels.

*Allium* peels amendments to soil considerably (*p* < 0.05) increased the WHC in comparison to the control. The percent of increase in WHC reached a maximum of 74% while a minimum of 25.1% upon amending soil with garlic peels and yellow onion peels in comparison to soil without peels **(**Fig. 2a**)**. The peels of onion and garlic represent a good source to extract fiber. The quantification of crude fiber content (Fig. 1) in various *Allium* peels indicated that garlic peels contained the maximum amount of 0.566 g/g DW, representing 44% of the total composition. To account for the increased RWC of the soil after amending it with various peels, a proportional correlation is observed with the fiber content of each peel (Fig. 2b).

### Effect of *Allium* peels on rice seedlings grown under water stress

**Fig. 3:**
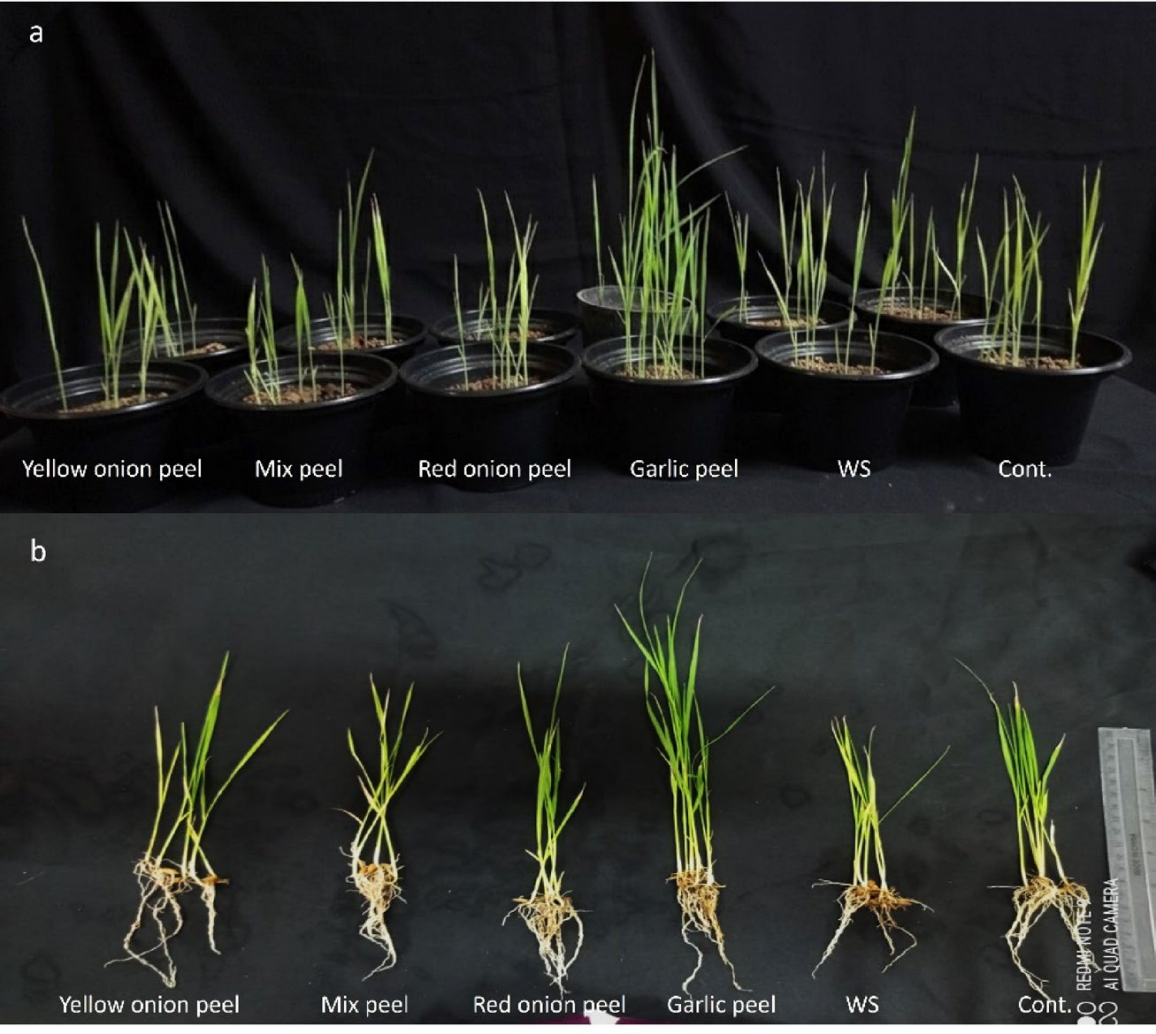
Effect of soil amendment with different *Allium* peels on rice growth. **a**: The effect on the shoot system, **b**: The effect on plant shoots and roots. The experiment comprises 6 different groups; each has three replicas: cont. (no treatment- the plants received 100 of % water field capacity), water stress (WS) in which the plants received 50% of water field capacity, and the other four groups, each one which was cultivated in soil amended with each type of the *Allium* peels independently, and all received 50% of water field capacity; the peels are: garlic peels, red onion peels, yellow onion peels, and a mix of 1:1:1 of each peel.

**Fig. 4:**
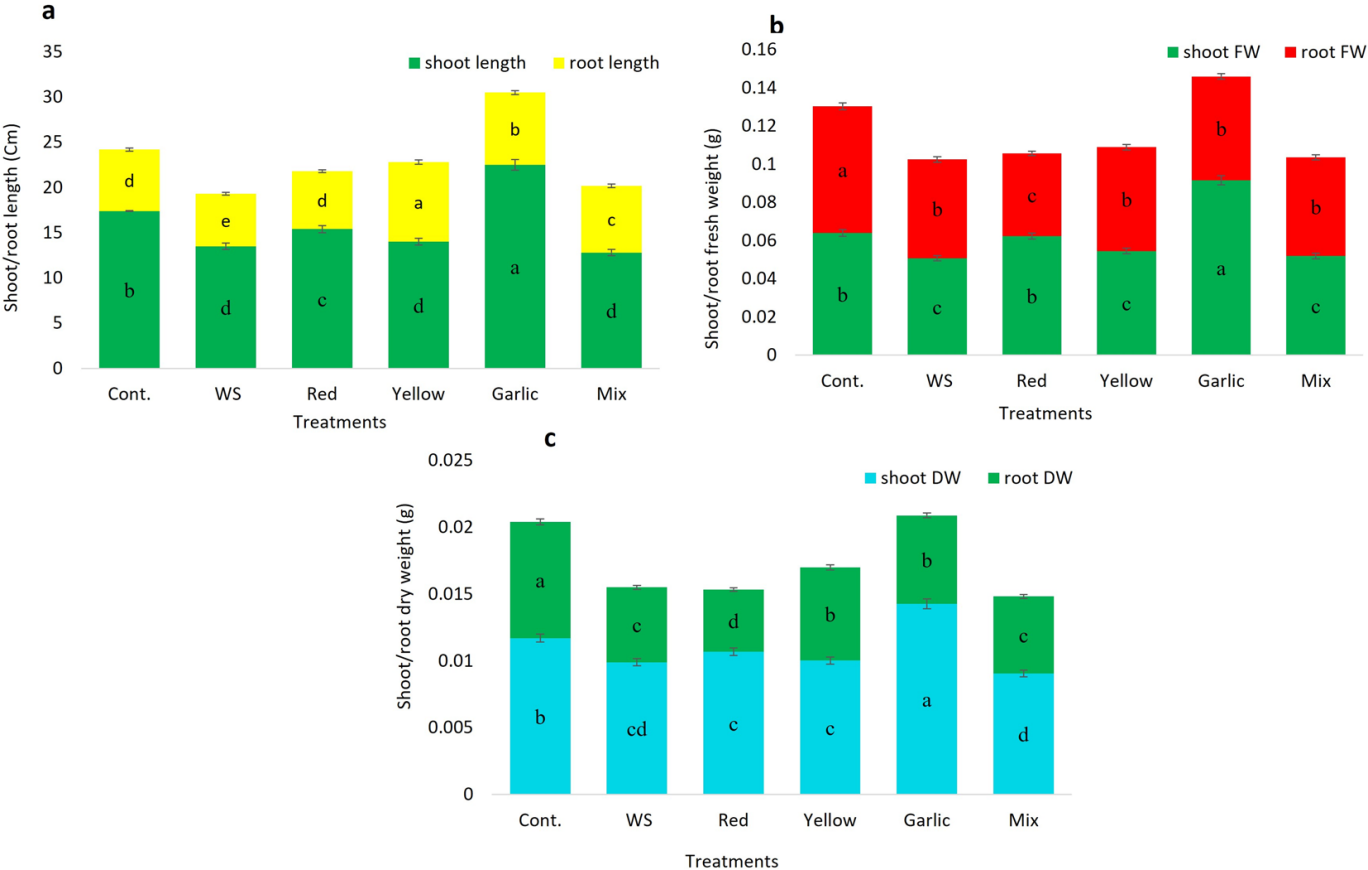
Effect of soil amendment with different *Allium* peels on rice growth. **a**: shoot and root length, **b**: shoot and root fresh weight, **c**: shoot and root dry weight. The data represent the average of six replicas, with error bars representing the standard error of the mean, and different letters show significant differences at *p* ≤ 0.05 according to One-Way ANOVA: Post Hoc Multiple Comparisons (Duncan).

The pot experiment of rice seedlings revealed significant growth decline when subjected to insufficient water, in which the percent of reduction was 22.41, 14.71, 20.64, 22.15, 15.38, and 35.63 % in the shoot and root length, fresh weight, and dry weight, respectively, in comparison to those of the controls. After amending the soil with different *Allium* peels, a positive enhancement in the measured vegetative growth attributes, even under a restricted water regime, was observed. The most pronounced positive effect was observed in the plants grown in soil amended with garlic peels. All the measured vegetative traits were significantly higher than control plants, confirming that the presence of the garlic peels has a stimulating influence on the rice plant growth at the vegetative stage. While amending soil with red onion peels showed a significantly promotive effect on the shoot and root length (cm), shoot fresh and dry weight (g) of 15.4 ± 0.407 cm, 6.4 ± 0.169 cm, 0.06234 ± 0.002 g, 0.011 ± 0.000283 g, respectively, relative to the control plants grown under water stress. On the other hand, amending soil with yellow onion peels or mixed peels significantly enhances root length, shoot, and root dry weight, reaching 8.8 ± 0.23 cm, 0.01 ± 0.0002 g, and 0.007 ± 0.00019 g, respectively, in the case of yellow onion peels in comparison to the control plants grown under water stress (Fig. 3, and Fig. 4).

### Physiological and biochemical attributes

#### Leaf photosynthetic pigments

In this study, a sharp decrease in photosynthetic pigments under water stress, where a decrease of 48.6% for chlorophyll a, 54% for chlorophyll b, and 53.9% for total pigments was observed as compared with the control. Nevertheless, outcomes in Table 3 revealed a significant difference (*p* ≤ 0.05) in the pigment fractions between water-stressed plants and *Allium* peels applied plants under this stress. Under water stress conditions, rice plants grown in soil amended with different peels (garlic, red, yellow onion, and their mix) significantly enhanced chlorophyll a (59.9, 59.6, 50.5, and 24.3% levels, respectively), compared with plants under water stress conditions without any amendment.

**Table 3:** Changes in pigment fractions (mg/g FW) of rice leaves affected by the application of peels of garlic, red and yellow onion and their mixture under water stress (WS, 50% field capacity) condition.

Treatments	Chl a	Chl b	Chl (a + b)	Carotenoids	Total pigments
Control	2.057 ± 0.063b	1.147 ± 0.03a	3.204 ± 0.093a	1.565 ± 0.041a	4.769 ± 0.134a
WS	1.257 ± 0.0279e	0.527 ± 0.0086e	1.784 ± 0.037e	0.810 ± 0.046d	2.594 ± 0.053e
Garlic peels	2.010 ± 0.069a	0.987 ± 0.026b	3.097 ± 0.095a	1.552 ± 0.041ab	4.549 ± 0.136ab
Red onion peels	2.007 ± 0.061b	0.908 ± 0.024c	2.914 ± 0.085b	1.449 ± 0.038b	4.363 ± 0.123b
Yellow onion peels	1.893 ± 0.055c	0.758 ± 0002d	2.651 ± 0.075c	1.188 ± 0.031c	3.839 ± 0.106c
Mix peels	1.562 ± 0.041d	0.889 ± 0.023c	2.451 ± 0.065d	1.121 ± 0.029c	3.571 ± 0.094d

Data are the mean ± standard error of six replicates. Means in the same column with the different letters are significantly different (*p* ≤ 0.05) according to Duncan’s multiple range test as determined by one-way analysis of variance (ANOVA).

#### Water status

**Fig. 5:**
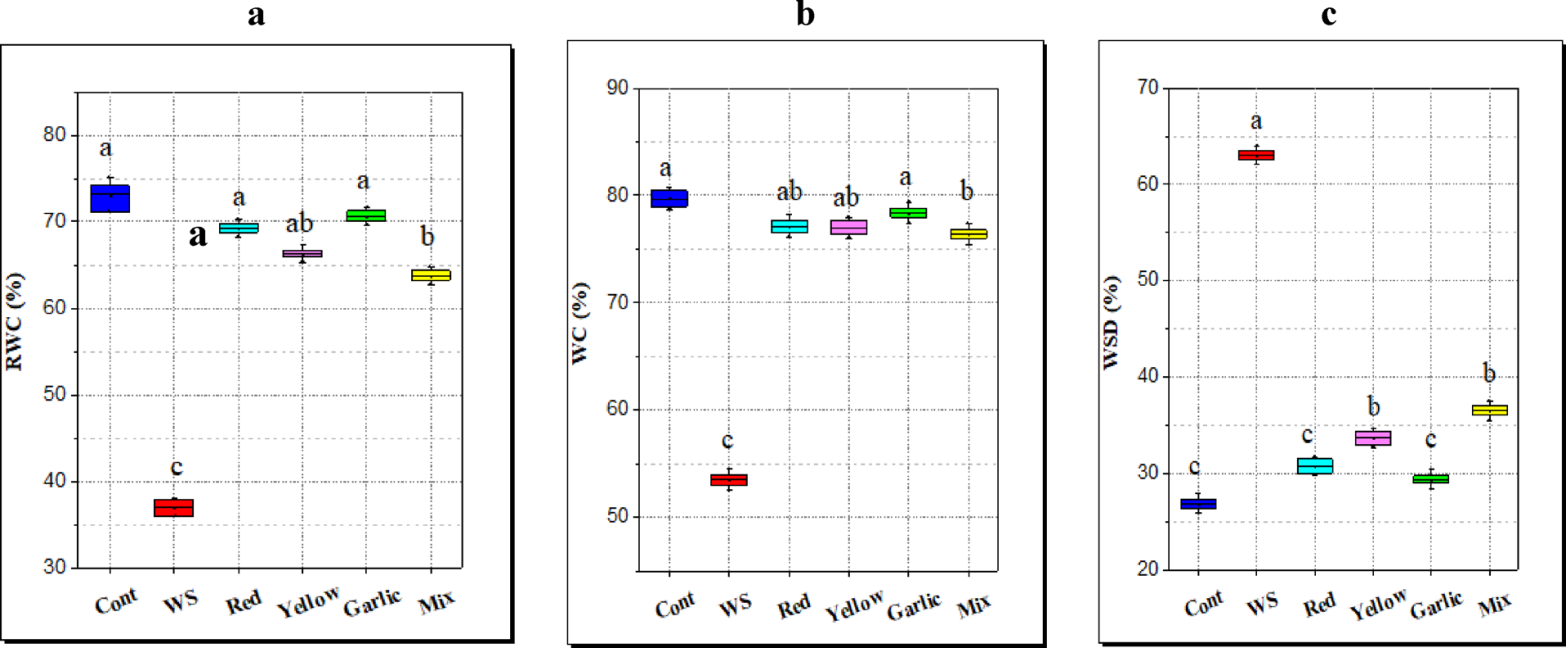
Effect of soil amendment with different *Allium* peels on water status of rice leaves under water stress (WS, 50% field capacity) condition. **a**: Relative water content (RWC), **b:** water content (WC), **c**: water saturation deficit (WSD). The data represent the average of six replicas with error bars representing the standard error of the mean, and different letters show significant differences at*p* ≤ 0.05 according to One-Way ANOVA: Post Hoc Multiple Comparisons (Duncan).

Water stress is one of the numerous variables that adversely impact the plant water status, results in Fig. 5 exhibits that water content (WC) and relative water content (RWC) show a subsequent decrease in rice plant leaves under water stress (32.9 and 49.5% of decrease) as compared with the control plant. However, water saturation deficit (WSD) increased with water stress (13.5% of increase). A remarkable result is that the application of *Allium* peels enhanced water status and increased WC and RWC, while decreasing WSD in rice plants under limited water. The rice plants grown in garlic peel-amended soil that was drought-stressed showed an increase of 49% and 91% in WC and RWC, while a decrease of 53% in WSD compared with control drought-stressed rice plants.

#### Impact on oxidative stress markers (LOX, MDA, and H_2_O_2_)

**Fig. 6:**
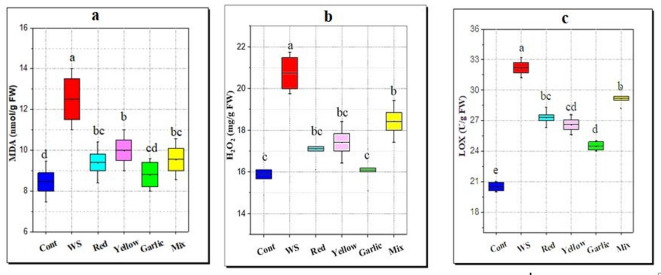
Effect of soil amendment with different *Allium* peels on stress marker of rice leaves under water stress (WS, 50% field capacity) condition. **a**: malondialdehyde content, **b**: H_2_O_2_ content, **c**: lipooxygenase activity. The data represent the average of six replicas, with error bars representing the standard error of the mean, and different letters show significant differences at *p* ≤ 0.05 according to One-Way ANOVA: Post Hoc Multiple Comparisons (Duncan).

As a biomarker of membrane lipid peroxidation, the MDA content significantly increased during water stress by 47.9% while an increase of 30.5% was found in H_2_O_2_ content as compared to non-stress conditions (Fig. 6). Nevertheless, the application of garlic and onion peels reduced the harmful effect of this stress on generation of ROS and as a result decreased the LOX activity, MDA and H_2_O_2_ contents. Among the different peels’ treatments, garlic peel application was the most effective in reducing LOX, MDA, and H_2_O_2_ by 24, 29, and 22% respectively, compared with water stress (Fig. 6).

#### Changes in proline and protein contents

**Fig. 7:**
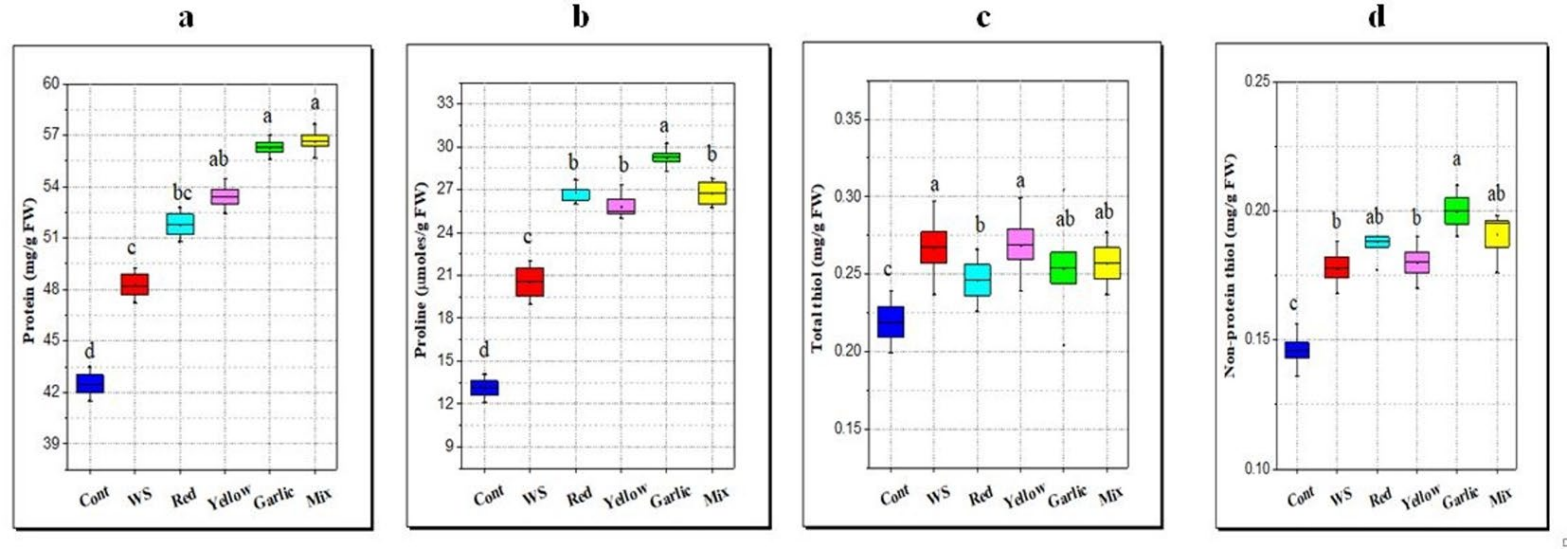
Effect of soil amendment with different *Allium* peels on biochemical parameters of rice leaves under water stress (WS, 50% field capacity) condition. **a**: protein content, **b**: proline content,**c**: total thiol, **d**: non-protein thiol content. The data represent the average of six replicas with error bars representing the standard error of the mean, and different letters show significant differences at*p* ≤ 0.05 according to One-Way ANOVA: Post Hoc Multiple Comparisons (Duncan).

In this study, as diagrammed in Fig. 7a and b, water stress elevated the proline and protein contents of rice leaves by 56.8 and 13.4% in comparison with the control. Furthermore, all peels application, especially garlic and red onion peel amendment, led to an additional rise in proline content by 42.5 and 31.4% compared to water stress (Fig. 7a). Also, diagrammed results in Fig. 7b showed that *Allium* peels application (garlic, red, yellow onion and their mix) caused a further increase in protein content by 32.4, 20.7, 25.6 and 33.2% and compared to control.

#### Total thiol and non-protein thiol

Due to the significant roles of thiols that are played in various physiological functions, in this study, thiol contents, both total thiol and non-protein thiol, were determined. Results of the present study (Fig. 7c and d) showed an increase in the content of total thiol and non-protein thiol (thiol-rich compounds other than proteins) in rice shoot under drought stress relative to the control. Also, data in Fig. 7 revealed that application of different *Allium* peels (garlic, red, and yellow onion) caused further and significant increases in non-protein thiol contents, while insignificant changes in the content of total thiol were observed. It was clear that the effect of garlic and red onion peels was the most effective treatment, since treatment of garlic peels increased non-protein thiol by 36.9% while red and yellow onion increased non-protein thiol by 28.8 and 23.3% compared with the control.

#### Antioxidant enzyme activity

**Fig. 8:**
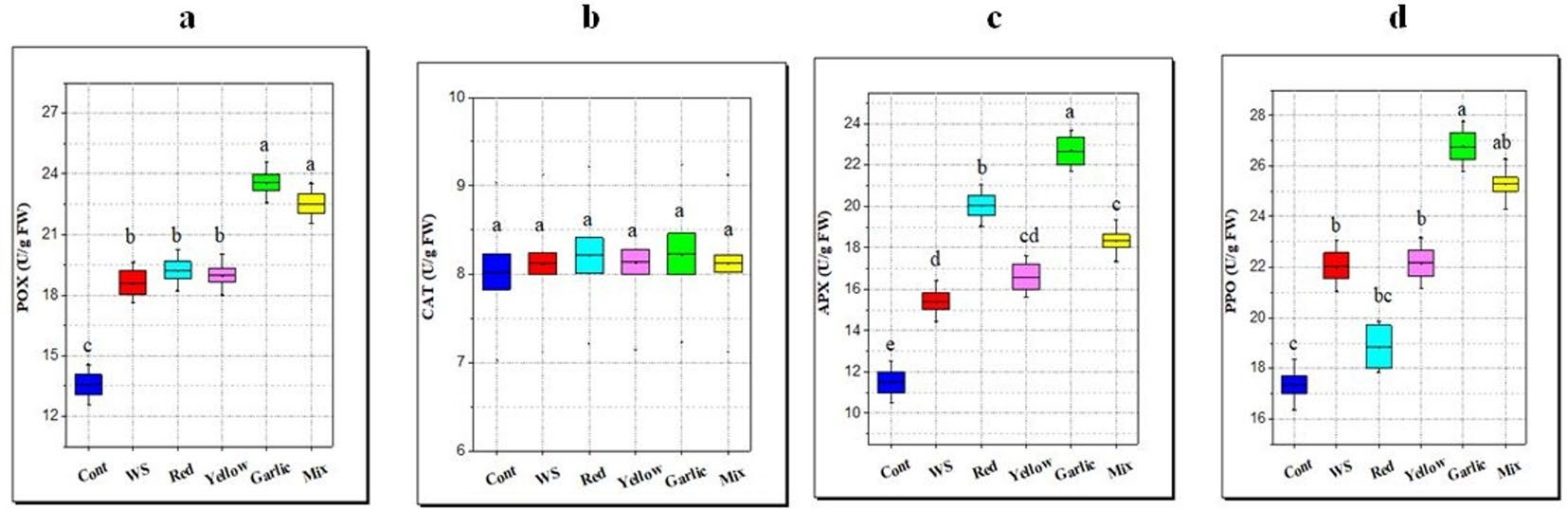
Effect of soil amendment with different *Allium* peels on antioxidant enzymes of rice leaves under water stress (WS, 50% field capacity) conditions. **a**: peroxidase activity (POX), **b**: catalase activity (CAT), **c**: ascorbate peroxidase activity (APX), **d**: polyphenol oxidase activity (PPO). The data represent the average of six replicas, with error bars representing the standard error of the mean, and different letters show significant differences at*p* ≤ 0.05 according to One-Way ANOVA: Post Hoc Multiple Comparisons (Duncan).

In this study, water stress increased POX, PPO, and APX activities by 37.3, 27.1, and 0.34.1%, respectively, compared with the control (Fig. 8). Moreover, applying different peels, especially garlic peel, further raised the activities of these antioxidant enzymes under water stress (Fig. 8). Results of the CAT did not show any significant difference between all the treatments.

### Effect of *Allium* peels on the genetic contents of rice seedlings

**Fig. 9:**
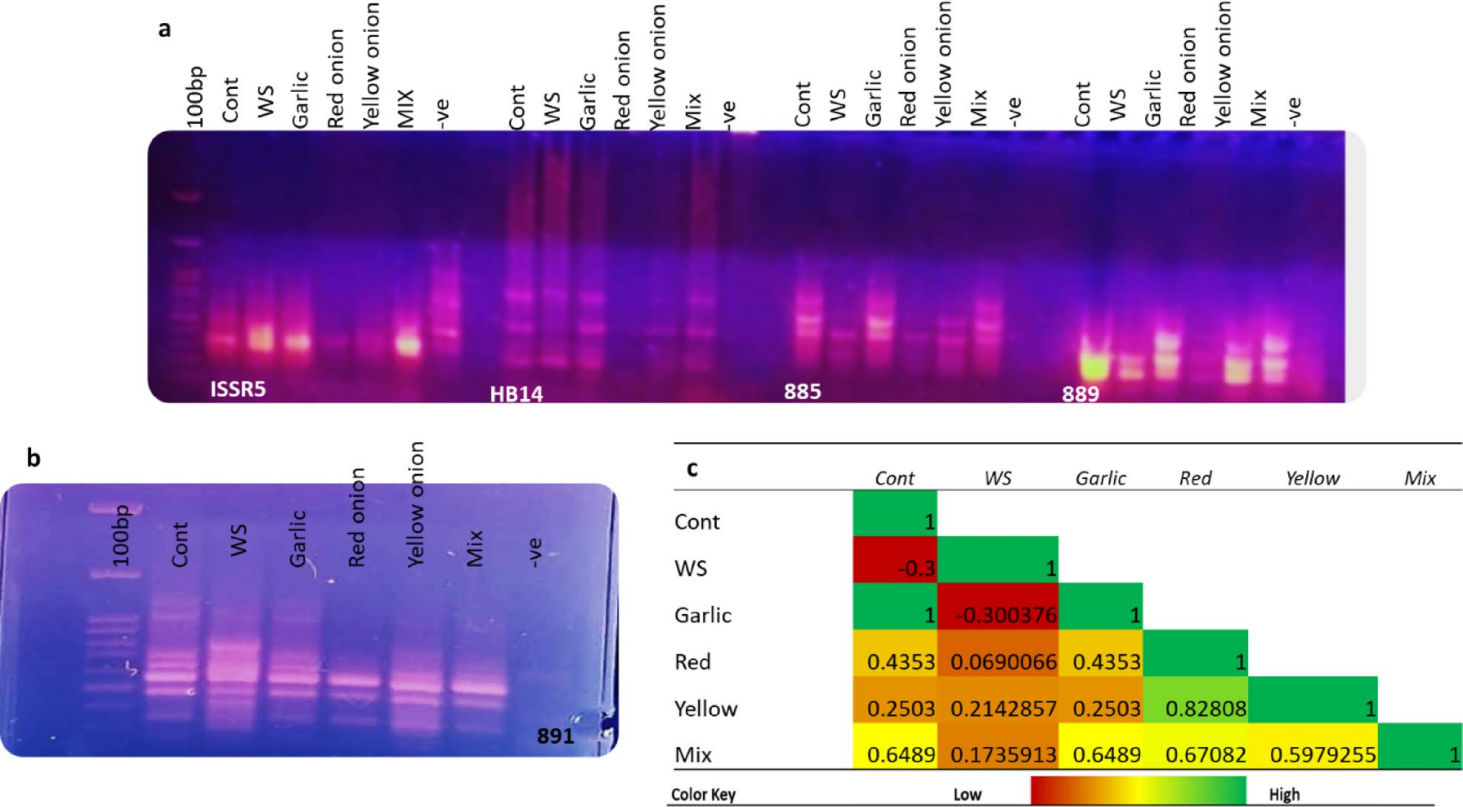
Effect of soil amendment with different *Allium* peels on rice plants’ DNA in response to different treatments by five different primers. **a**: shows the electrophoretic pattern for ISSR5, HB14, 885, and 889 primers. **b**: shows electrophoretic pattern for 891 primers. 100 pb refers to the DNA ladder, -ve refers to the negative control, and no amplification confirms no PCR contamination. **c**: correlation analysis among different treatments based on the ISSR data.

To investigate whether the alterations noticed in the vegetative growth, physiological, and biochemical parameters of rice seedling upon planting in soil enriched with *Allium* peels were combined with alterations in the genetic material, five ISSR primers were used. All the primers generated an overall 22 consistent markers **(**Table 2; Fig. 9a, b**)**. The amplified markers varied in size from 860 to 150 base pairs, with a total variation among treatments of 63.6%. Primer I-891 produced the highest number of nine markers, representing 40.9% of the total markers generated. Nineteen different alleles were amplified from control rice seedlings, in which it was reduced to 14 alleles for drought-stressed plants. On the other hand, amending soil with garlic peel even under a restricted water regime causes retention of the genetic content to the level detected in the control plants of 19 markers. While amending soil with red onion or mixed peels caused genetic alteration that resulted in the 12 and 16 ISSR markers. The correlation of the genetic matrix revealed a strong positive correlation between control, garlic, and red onion peels amended soil. While strong negative correlation was observed between control, water-stressed control, and garlic peel amended soil (Fig. 9c).

### Data inter-relationship analysis

**Fig. 10:**
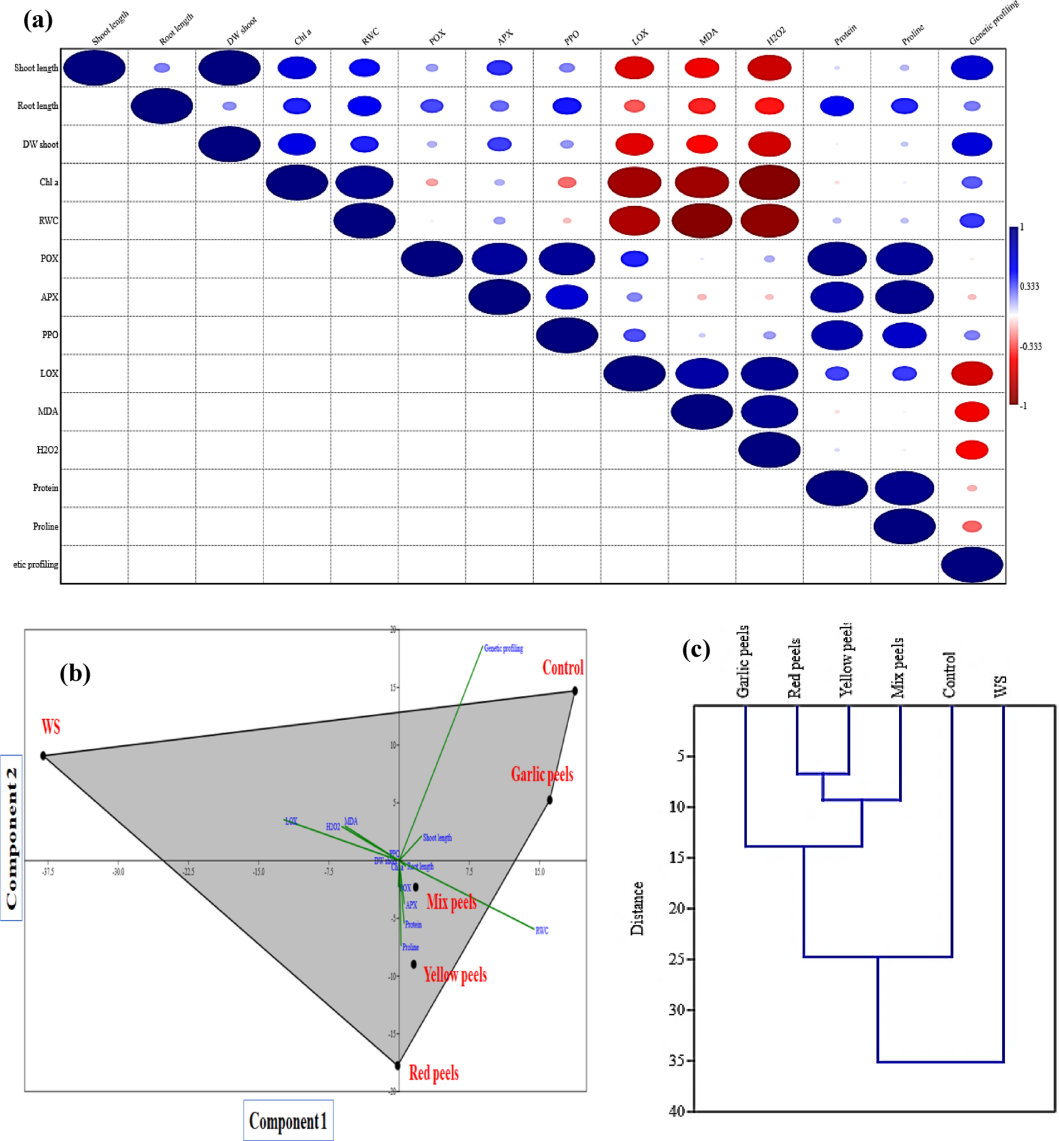
Data inter-relationship analysis, **a**: Pearson correlation between different parameters, **b**: Scatter plot of principal component analysis (PCA) analysis between different parameters and treatments (biplot), and **c**: Hierarchical clustering analysis (HCA) between different treatments.

Pearson correlation, PCA, and HCA were conducted to better understand the results (Fig. 10a-c). Pearson correlation between different measured parameters (Fig. 10a) revealed a strong positive correlation between RWC with growth parameters, and Chl a. On the contrary, a significant negative correlation was found between stress markers (H_2_O_2_, MDA, and LOX) and most of the measured parameters, besides the genetic profiling. On the PCA-associated scatter plot, we observed a strong separation between the treatments in terms of PC1 (62.8%) and PC2 (22.1%) (Fig. 10b). Furthermore, the PCA-associated loading plot indicated that shoot length, RWC, protein and genetic profiling more positively correlated with applying peels especially garlic peels, whereas stress markers (H_2_O_2_, MDA and LOX) were associated with WS (plants grown under water stress conditions). The HCA, which demonstrated the differences among treatments, agreed with the PCA results (Fig. 10c).

## Discussion

### *Allium peels*: a physicochemical examination

*Allium* peel chemical composition can vary with species, cultivar type, and storage time; as a result, the components of target compounds must thus be ascertained on a case-by-case basis. The bulk components of the *Allium* peels utilized were fibers and ashes. Also, *Allium* waste has been known as a source of antioxidant compounds, fiber, carbohydrates, and polyphenols, which opens possibilities for its further employment as a source of active compounds functional in food applications, antimicrobial, anti-inflammatory, antioxidant, and immunomodulatory^13,56^. Antioxidants are the bulk active ingredient detected in the garlic peels, and considerable amount of phenolics and flavonoids was also detected, the least in garlic and the highest in red onion peels. This may be a result of anthocyanins, found in the red onion variety^56^. Main flavonols in onion are quercetin and its glucoside derivatives, which have several biological activities, including antioxidant, anti-inflammatory, anticancer properties, and prevention of cardiovascular diseases^57–59^. Organosulfuric compounds in onions are carriers of their specific aroma and flavor. The higher amount of sulphur was detected in red onion peels, representing 14% while only 0.56% was detected in garlic peels. According to a recent study, organosulfur compounds derived from *A. cepa* have a strong antimicrobial and pesticidal effect against the vast majority of phytopathogens tested in soil, making them a promising tool for better crop pest management^12^.

### Effect of *Allium* peels on the water holding capacity (WHC) of the soil

Applying soil amendments with peels can enhance the physical characteristics of the soil by increasing its capacity to hold water (increase WHC), which will boost crop output. This can reduce the demand for irrigation and increase crop yield in semi-arid areas with little rainfall. *Allium* peels mixed with sandy soil may create smaller pores, which can retain more water^16^. Similarly, amending sandy soil with clay leads to an increase in the plant available water, and the water retention capacity increases in proportion to the amount of clay added^60^. In addition, amending soil with *Allium* peels is environmentally safe as it represents a new route to recycle agricultural waste for increasing WHC of the soil as well as providing a source to increase soil fertility.

### The relation of *Allium* peels’ crude fiber content and WHC of the soil

The quantity of plant fiber has a significant impact on the WHC. Plant fibers consist of cross-linked microfibrils of cellulose molecules within the cell wall. Plant fibers can bind large amounts of water due to the strong hydrogen bonding between water molecules and the hydroxyl groups on the cellulose chains. Moreover, the fibrillation process, which dissects fibers into finer fibrils, increases the available surface area significantly^61^. The soil’s water retention capacity is further improved through the increased surface area, which facilitates hydrogen bonding between water molecules and the cellulose fibrils^62^. In a recent study, a fibrous composite was synthesized based on cellulose microfibril material *via* the emulsion polymerization method. The resulting fibrous composite showed the best water absorption qualities while maintaining its structure, even after 24 h of floating on water^63^.

### Effect of *Allium* peels on rice seedlings grown under water stress

Water stress reduces cell turgor because it restricts the amount of water available in the soil, which inhibits cell division and expansion, and lowers plant growth and development^64,65^. In accordance with other studies such as El-Okkiah et al.^65^ study, the pot experiment of rice seedlings revealed significant growth decline when subjected to insufficient water. On the contrary, all the measured vegetative traits of rice plants grown in peels-amended soil, even under a restricted water regime, were significantly higher than stressed control plants. This effect was more pronounced for plants grown in garlic peels amended soil, confirming that the presence of the garlic peels has a stimulating influence on the rice plant growth at the vegetative stage. This observation could be attributed to the concomitant increase in RWC upon soil amending with the peels. When plants are stressed by drought, their RWC and leaf water potential drop^66^. Foliar spray of silica improves water status in rice and other crops under drought conditions^65^. Also, amending sandy soil with bentonite-humic acid mixture improved the physical characteristics of the soil, growth, and yield of maize plants^67^.

### Effect of *Allium* peels on physiological and biochemical attributes of rice seedlings grown under water stress

#### Leaf photosynthetic pigments

The primary building blocks of photosynthesis are plant pigments, particularly chlorophyll, which is needed for light absorption and the production of reducing power. Carotenoids, along with other pigments, are crucial for photoprotection and serve as a precursor to signals that guide plant growth in stressful environments^68^. The present results indicated a sharp decrease in pigment fractions under drought stress. Consequently, Hussain et al. reported that water deficiency leads to disrupted flow of water towards another cell from xylem, including lesser turgor pressure and, consequently, poor cell development and reduced leaf area in crops^66^. Due to the restricted water potential during drought stress, leaf growth is decreased. Water stress modifies both the typical rate of photosynthesis and the properties of plant gas exchange^69^. Limited water condition leads to stomata closure, reducing carbon dioxide influx to leaves, decline of turgor pressure, and driving extra electrons for the formation of reactive oxygen species (ROS)^69–71^. Water stress damages the normal functions of photosystem I and II^72^. Both photosystems are essential in the reduction reaction and the production of ATP. Photosynthetic capacities of leaves and water availability to the root zones are crucial elements that lessen yield in sensitive rice genotypes under drought stress conditions in rice^69^.

The enhancement of chlorophylls in rice leaves grown in soil amended with different peels might be due to an improvement of the plants’ capacity to absorb nutrients and water, both of which are necessary for the production of pigments used in photosynthetic processes. This could be as a result of that garlic is rich in complex organic sulphides, and onion is rich in carbohydrates, flavonoids, and phenols, and this is confirmed in our analysis of these peels^73^ (Fig. 1) As well, garlic peels are a good nutrient source for plant growth as they are rich in vitamins, antioxidants, and nutritional value like fiber, calcium, calories, iron, potassium, and magnesium. Onion and garlic peels, biowaste, can be utilized as a natural growth enhancer in a sustainable approach for developing microgreens of fenugreek and falooda seeds^74^.

#### Water status

Plant and water relationship can be represented in a variety of ways, such as a leaf’s water potential and relative water content (RWC)^70^. According to Gupta et al.^75^, RWC is a crucial component of plant water relations and is regarded as the most accurate way to measure the overall water status of plants. It reflects changes in the plants’ turgor and water potential. Drought stress affects plant water status^76,77^ by decreasing water availability, reducing leaf water potential, transpiration rate, and increasing leaf temperature, because the drought has a detrimental influence on the processing of stomatal opening and closure. All these parameters decline under water stress in rice^78,79^.

Interestingly, the more RWC and WC in rice grown in soil amended with different peels, especially *Allium* peels, could be explained by the capacity of these peels to maintain water and facilitate its uptake and absorption by rice roots. This outcome is in line with that of El-Serafy et al.^80^, who confirmed that the application of fruit peels exhibited an enhancement in RWC of *Schefflera arboricola* leaves. As well, Mi et al.^16^, revealed that bentonite mixing with sandy soil may create smaller pores, which can preserve more water. Similarly, adding more clay to sandy soil increases plant availability of water and increases water retention capacity proportionately^60^.

#### Impact on oxidative stress markers (LOX, MDA, and H_2_O_2_)

Disturbance between ROS production and quenching is a common consequence of water stress. Superoxide radicals, hydroxyl free radicals, H_2_O_2_, and singlet oxygen are examples of ROS. They can induce cellular oxidative damage, denaturation of proteins, peroxidation of lipids, and DNA mutation. They can also upset cellular homeostasis^81,82^. Under water-stressed situations, exclusion of water from the membrane induces damage and dislocation of the normal lipid structure, and consequently, the membrane becomes permeable during desiccation. Behaviors of cell membrane stability/membrane stability index have been used to determine its correlation with the yield of rice under drought stress^83^. In plants, increased lipid peroxidation (MDA), LOX activity, electrolyte leakage, and H_2_O_2_ production have been used to assess the increase in ROS production under drought^84^ as indicated in this study. On the contrary, the decrease in stress markers in rice seedlings with peels amendment might be attributed to the content of phenolics, flavonoids, and antioxidants present in these peels **(**Fig. 1**)**, which could be reflected in rice seedlings. These outcomes concur with El-Serafy et al.^80^, where higher decreases in MDA and H_2_O_2_ levels were shown in *S. arboricola* plants, along with less ion leakage and better maintenance of membrane functions after applying fruit peels to these plants grown under hot conditions, indicating the function of these peels as antioxidants.

#### Changes in proline and protein contents

As water deficit occurs, plants store a variety of organic and inorganic solutes in their cytoplasm to decrease osmotic potential and preserve cell turgor^85^. Osmotically active substances known as compatible solutes, such as proline, protein, soluble carbohydrates, and free amino acids, accumulate in high quantities in the cytoplasm to produce osmotic adjustment and enhance water intake from drying soil Abdelhameed et al.^86^. According to Vajrabhaya et al.^87^, proline functions as an osmolyte and accumulates to improve performance and drought resistance. In agreement with the present results, Lum et al.^88^, revealed that when rice is subjected to drought stress, changes in the concentration of proline have been seen. Proline serves as a great osmolyte and is also involved in three important processes during stress: metal chelation, antioxidant defense, and signaling^89^. Proline buildup during drought stress may enhance the plant’s capacity to repair damage by raising antioxidant activity. Proline content rises greater than that of other amino acids in plants under water stress, and this phenomenon has been utilized as a biochemical marker to choose cultivars intended to withstand such circumstances^90^. When plants under water stress were subjected to orange peel extract or ascorbic acid treatment, the amount of free amino acids significantly increased in comparison to the control plant^91^.

Our results of increasing protein content with peels application align with those of El-Serafy et al.^80^, who attributed the increase in protein content using fruit peels to reprogramming gene expression, resulting in the synthesis of new proteins and more storage proteins that help plants to grow and develop. Additionally, they showed that this substance is crucial for cells to adapt to a variety of unfavorable environmental conditions by increasing the cytoplasmic osmotic pressure, stabilizing proteins and membranes, and preserving the relatively high-water content necessary for plant growth and cellular processes.

#### Total thiol and non-protein thiol

The present results showed augmentation of total thiol and non-protein thiol under drought stress and peels application. These findings are aligned with the study of Dey et al.^92^, who revealed an accumulation of non-protein thiol compounds in flag leaves of rice cultivars during drought stress. Another study by Bhoomika et al.^91^ revealed a rise in non-protein thiols in seedlings of rice cultivars differing in aluminum tolerance. Biothiols are incredibly effective antioxidants that shield cells from the damaging effects of free radical injury due to their capacity to interact with free radicals. Because of this, thiols’ antioxidant qualities have received the majority of attention. Some non-protein thiols such as glutathione serve as key non-enzymatic antioxidants in plants, which either directly or indirectly (as a substrate of enzymes) scavenge several potentially toxic ROS, such as O_2_^•−^ or ^•^OH, and help cells in coping up with oxidative stress brought about by a variety of stressors^93^.

#### Antioxidant enzyme activity

Enzymatic and non-enzymatic antioxidants work together to form a complex antioxidant system that shields plant cells from the detrimental consequences of ROS. The expression of these antioxidants can increase rice’s resistance to drought since they are essential parts of the plant’s ROS scavenging mechanism^94^. With rising levels of drought stress in rice, the activities of glutathione and APX ^95^, phenylalanine ammonia lyase, and CAT^96^ consistently upsurge. Increases in the activities of these antioxidant defense enzymes indicate rice’s preventive activity against oxidative damage induced by drought. The activities of superoxide dismutase, POX, and CAT have been shown to efficiently reduce ROS, which in turn lessens the detrimental effects of drought^88^. Rice seedlings that have been preconditioned to a mild drought respond differently to antioxidant enzymes, which helps them adapt to an intermediate drought stress environment more successfully^97^.

Thus, one method for lessening or preventing oxidative damage and enhancing plants’ tolerance to water stress may be to improve the naturally occurring antioxidant components (enzymatic and non-enzymatic)^98^. Mohamed et al.^99^, showed that the application of orange peels caused significant increases in CAT and POX activities in quinoa leaves as compared to control plants. POX and CAT play an essential role in scavenging H_2_O_2_ toxicity. The combined action of CAT and SOD converts the toxic O_2_^•−^ and H_2_O_2_ to water and molecular oxygen, thus averting the cellular damage under unfavorable conditions like water stress^100^. Collectively, rice plants grown in peel-amended soils are less stressed, and they increase certain parameters that increase during stress (proline, total thiol, non-protein thiol, and POX and APX activities) compared to plants grown under water stress (WS). Perhaps it is because the beneficial effect of *Allium* peels is not only to retain water but also to trigger defense systems.

### Effect of *Allium* peels on the genetic contents of rice seedlings

The plant’s response to the surrounding environment would be determined by the extent of genetic alteration present in the plant. Because ISSR is a multilocus technique that spans randomly distributed microsatellite regions throughout the genome. So, it can be applied in studies involving genetic identity and gene mapping^55,101^. Altered genetic profiling was documented in rice plants grown under a restricted water regime compared to controls; this finding underlines the effect of drought on the genetic content. An ISSR profile was employed to determine the genome template stability of *M. parviflora* plants under drought stress, and the results confirmed that the drought reduced the GTS and caused an alteration in the ISSR profiling Abdelhameed et al.^86^. The appearance and disappearance of ISSR marker bands may explain the changes in the physiological and biochemical parameters of rice plants in response to water stress in different soil mixes. Plants exposed to water stress produce ROS that may target DNA and nuclear proteins, causing its degradation, and interfering with the cell cycle, resulting in genome modification, thereby compromising the DNA repair mechanisms^102^. Interestingly, oxidative stress markers such as MDA and H_2_O_2_ were elevated in water-stressed plants. On the other hand, mixing soil with garlic peel decreased these stress markers. It has previously been proposed that phenols have a triple synergistic impact in scavenging ROS, mending DNA, radicals, and metal chelation^103^. Also, the current results show significant elevation in the level of non-protein thiols, POD, PPO, and APX activities, were are all efficient antioxidants that work to shield the cells from the effects of harm brought on by free radicals^93^ which may be in favor of maintaining genome integrity. Previous research has demonstrated that drought stress affects genome stability and causes genetic variation in the grassland species *Bouteloua eriopoda*^104^. The positive correlation between control, garlic, and red onion peels in amended soil. While a negative correlation was observed between control, water-stressed control, and garlic peel amended soil, it suggested that the observed changes in growth, physiological, and biochemical attributes are based on a genetic basis.

## Conclusions

Considering the above findings, water stress caused growth reduction, oxidative damage, and alteration in the genetic profiling of rice through excessive generation of ROS (high MDA and H_2_O_2_ content and LOX activity). Proper addition of *Allium* peels could improve the growth of rice plants under water stress through enhancing proline, non-protein thiol, the antioxidant systems, and water content, and retain genome integrity. This effect may be due to the ability of *Allium* to maintain RWC of the soil under water stress. In addition, the analysis of the peels revealed the presence of various organic compounds that may increase nutrient availability in the soil and thus enhance rice vegetative growth, which would ultimately enhance yield. Additional investigation is needed to explore the long-term effects of these amendments on soil fertility and different crops in field conditions.

## Limitations and future perspectives

While the present study demonstrates that *Allium* peel amendments improve rice vegetative performance under limited irrigation through enhanced morphological, physiological, and biochemical responses, one limitation is the absence of crop yield data. Because the experiment was conducted under controlled pot conditions, reliable yield estimation was not feasible. Instead, this work focused on mechanistic parameters to clarify how peel amendments influence soil water holding capacity and rice stress tolerance. Nevertheless, yield is a critical agronomic trait, and future field-scale experiments will be conducted to validate these findings under realistic cultivation conditions and to determine the long-term effects of *Allium* peel amendments on rice productivity and how these amendments would alter gene expression profiling.

## Supplementary Information

Below is the link to the electronic supplementary material.

Supplementary Material 1

## Data Availability

All data have been provided with the manuscript.

## Abbreviations

APX: ascorbate peroxidase
BSA: bovine serum albumin
CAT: catalase
DW: dry weight
FW: fresh weight
GAE: gallic acid equivalent
ISSR: Inter-Simple Sequence Repeats
LOX: lipoxygenase
MDA: malondialdehyde
OC: organic carbon
POX: peroxidase
PPO: polyphenol oxidase
PCR: polymerase chain reaction
QE: quercetin equivalent
ROS: reactive oxygen species
RWC: relative water content
WC: water content
WHC: water holding capacity
WSD: water saturation deficit
WS: water stress
WS: water shortage
