# Supplementary figures and images for: Utilization of Allium peels to improve soil water holding capacity and rice growth under water stress conditions
Source: Sci Rep. 2025 Nov 25;15:41830. doi: 10.1038/s41598-025-27336-8 (PMC12647662; doi:10.1038/s41598-025-27336-8)

Full-length gels and blots


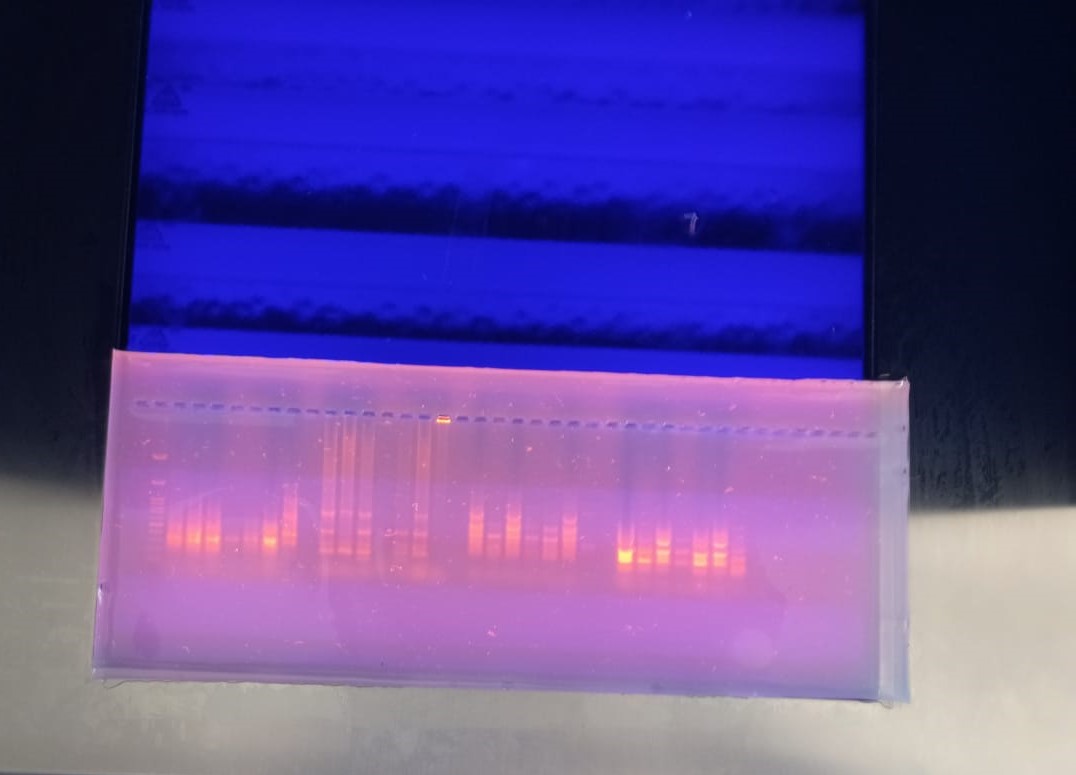


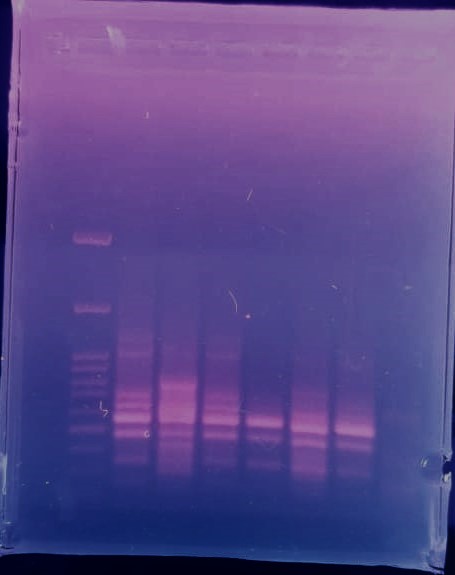

Supplement: Supplementary file 1 — Supplementary Material 1 [file 41598_2025_27336_MOESM1_ESM.docx]
